# Sinonasal mucosal melanoma presenting as a maxillary sinus mass: A case report

**DOI:** 10.1002/ccr3.5374

**Published:** 2022-02-01

**Authors:** Kamal Gautam, Sansar Babu Tiwari, Suraj Shrestha, Prabin Gaire, Malati Dulal, Bibek Man Shrestha, Roman Dhital, Usha Manandhar, Arun Kumar Baral

**Affiliations:** ^1^ Oxford University Clinical Research Unit Patan Academy of Health Sciences Kathmandu Nepal; ^2^ Department of Pathology Tribhuvan University Teaching Hospital Maharajgunj Medical Campus Kathmandu Nepal; ^3^ Maharajgunj Medical Campus Institute of Medicine Kathmandu Nepal; ^4^ Department of ENT Tribhuvan University Teaching Hospital Maharajgunj Medical Campus Kathmandu Nepal; ^5^ Nepal National Hospital Kathmandu Nepal; ^6^ Shivanagar Primary Health Care Center Chitwan Nepal

**Keywords:** intranasal melanoma, maxillary sinus, radiotherapy

## Abstract

Maxillary sinus melanoma is a rare mucosal melanoma difficult to diagnose in the absence of pigmentation. Intranasal masses presenting with the features of occult malignancy and rapid progression should always be investigated in the line of melanoma irrespective of pigmentation. The histopathological and immunohistochemical examination helps to confirm the diagnosis.

## Graphical abstract

Maxillary sinus melanoma is a rare mucosal melanoma difficult to diagnose in the absence of pigmentation. Intranasal masses with rapid progression should always be suspected and investigated in the line of melanoma irrespective of pigmentation. The histopathological and immunohistochemical examination helps to confirm the diagnosis.

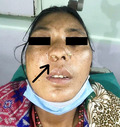

## 1 INTRODUCTION

Mucosal melanoma is a malignant neoplasm arising from melanocytes in the mucosa. Sinonasal mucosal melanoma accounts for 1% of all the melanomas encountered and 4% of all the sinonasal tumors.^1^ It is an uncommon finding with a reported incidence of 0.3 cases per million people per year but it accounts for approximately 25% of all melanoma in the Asian population.^2^ Most patients present with sinonasal congestion and epistaxis. It is highly aggressive due to its rich vascularity and tends to metastasize. It poses a diagnostic challenge as around 20% are multifocal and around 40% are amelanotic.^2^

Here, we report a case of a 33‐year‐old female patient who presented with right nasal cavity mass and features of nasal obstruction for one and half months which on histopathological and immunohistochemical examination were suggestive of a sinonasal mucosal melanoma.

## 2 CASE REPORT

A 33‐year‐old non‐smoker female patient presented to the outpatient department with right nasal obstruction for one and half months, which was initially partial, gradually progressed to complete along with visualization of mass in the right nasal cavity. She also complained of blood‐tinged right nasal discharge and right hemifacial pain for the same duration. The patient has lost 5 kg over 2 months and does not have any family history of malignancy.

**FIGURE 1:**
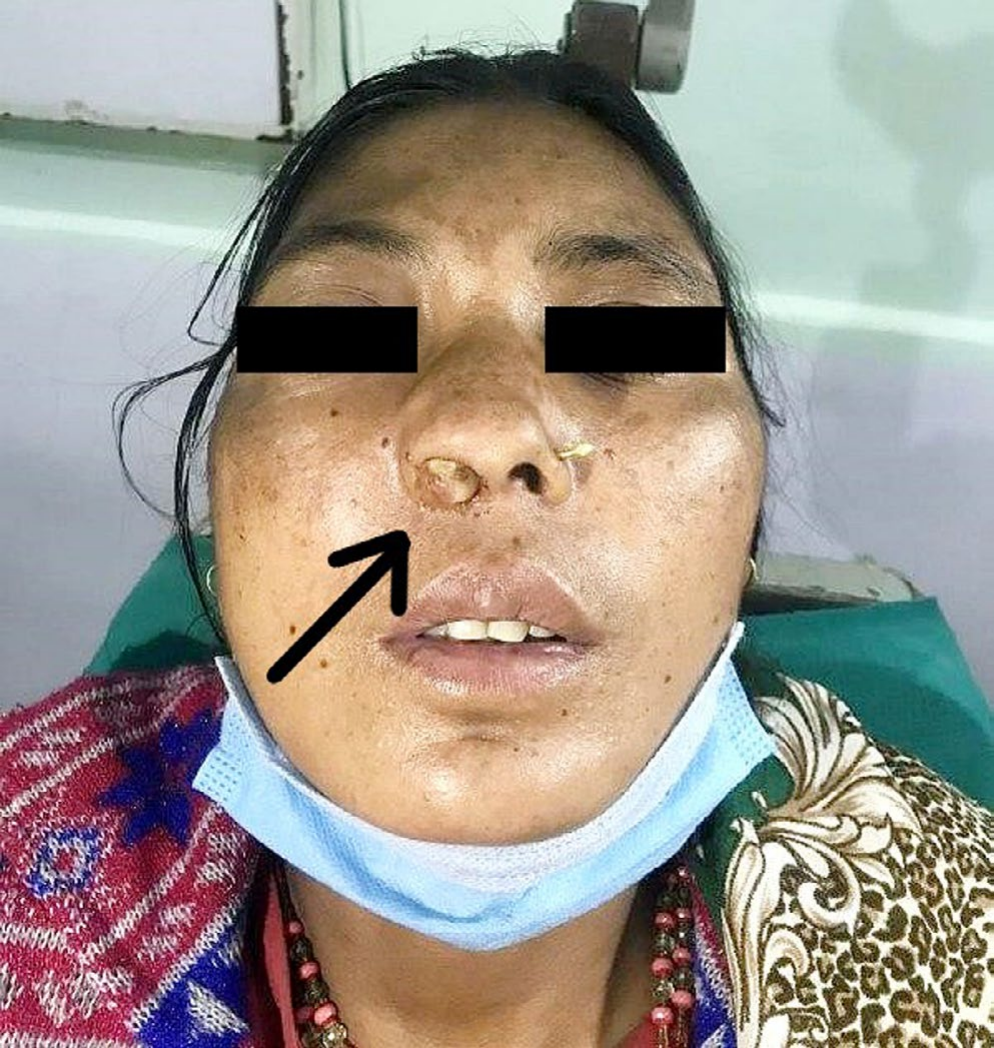
Right nasolabial fold obliteration and mass reaching up to the right nostril

On examination of the nose, externally, the right nasofacial groove was obliterated, and on nasal vestibular examination, grayish‐white mass was visualized occupying the whole of the right nasal cavity with the lowermost part of mass reaching just up to the nostril. (Figure 1).

**FIGURE 2:**
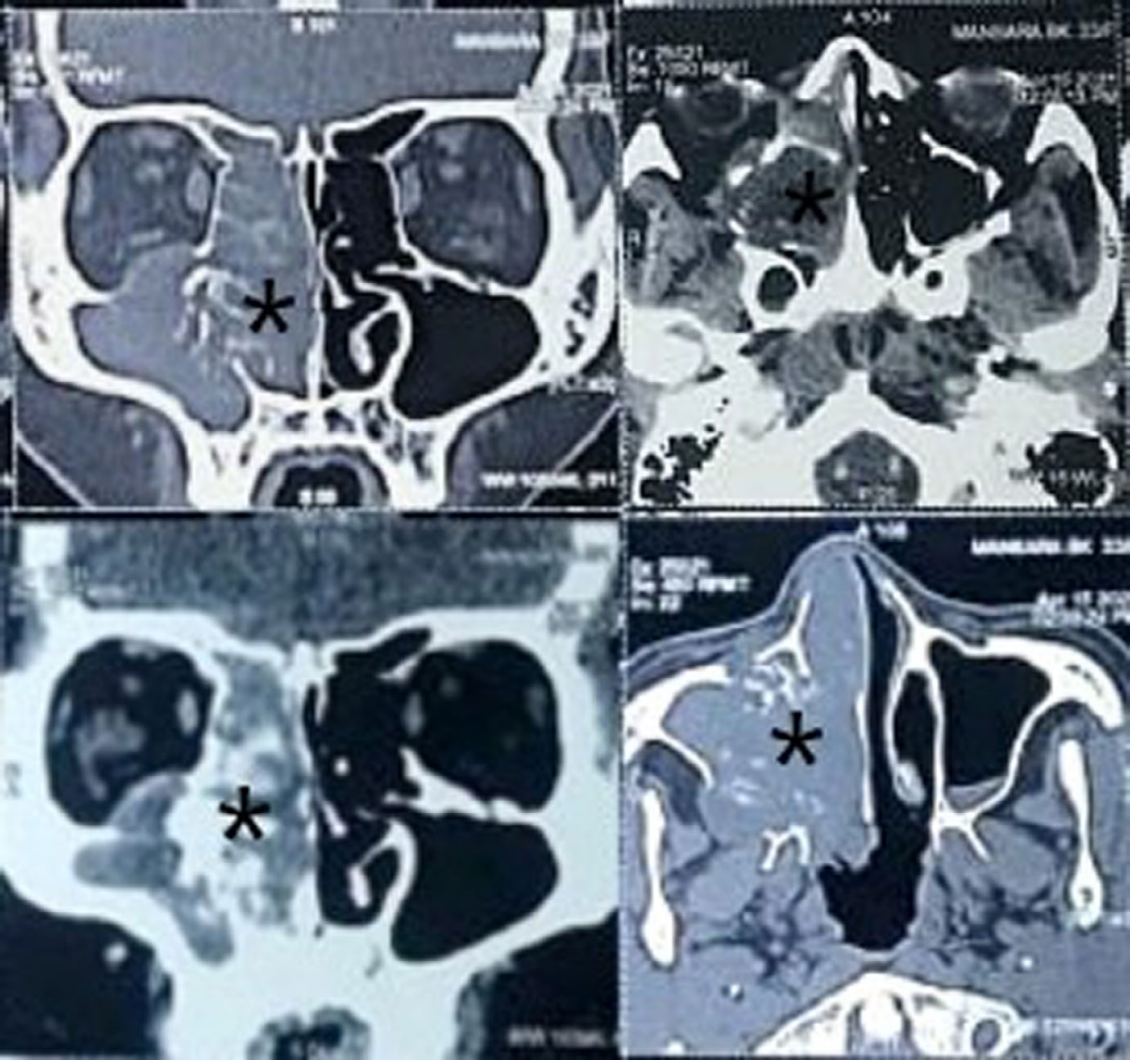
CT nose and PNS demonstrating soft tissue opacification in the right maxillary sinus, ethmoid and frontal sinus along with the erosion of surrounding bony structures

On blood investigation, hemoglobin level was 11 g/dl, and all other parameters were within normal limits. CT scan of nose and paranasal sinuses showed enhancing soft tissue opacification in the right maxillary sinus, ethmoid and frontal sinus extending through the eroded and widened osteo‐meatal complex medially into the nasal cavity with multiple hyperdensities. Also, the mass was eroding the maxillary walls, right lamina papyracea, cribriform plate, and extending up to the right orbital cavity and right infratemporal fossa. (Figure 2). Punch biopsy was done under local anesthesia which yielded multiple pieces of grayish white tissues without melanin pigmentation. Histopathological examination showed sheets of singly dispersed tumor cells with scant cytoplasm, coarse chromatin, and prominent nucleoli without necrosis along with focal cartilaginous changes.

**FIGURE 3:**
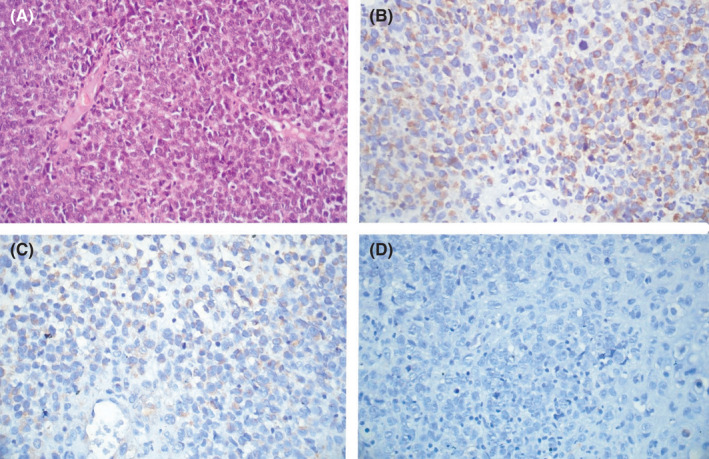
(A) Sheets of dispersed tumor cells with a scant amount of cytoplasm and coarse chromatin. (B) Tumor cells show moderate diffuse cytoplasmic HMB‐45 positivity, (C) weak vimentin positivity, and (D) negative CD45 immunostaining

Immunohistochemical studies showed strong and diffuse HMB‐45 and weak vimentin positivity. The tumor cells were negative for S100, CD45, cytokeratin, and desmin. (Figure 3) With these features, a diagnosis of sinonasal mucosal melanoma was made. The patient is currently undergoing radiotherapy. Surgical removal of the tumor is planned; however; the patient is reluctant to undergo surgery due to economic constraints. She is planned to be evaluated after 3 months.

## 3 DISCUSSION

Mucosal melanoma of the head and neck region is an uncommon entity and accounts for 1% of all melanomas without sex predilection.^3^ The entity is common in the seventh decade of life. Most of the mucosal melanomas arise from the melanocytes in the lining epithelium. Sinonasal mucosal melanomas have a possible association with melanocytosis, which is seen in uveal melanomas. The nasal cavity is the most common site followed by the septum and nasopharynx. However, the maxillary sinus is the least encountered site.^4^ Our patient is an adult female of age 33 and had a right maxillary sinus mass.

Although formaldehyde exposure and tobacco smoking are considered to play a role in pathogenesis, the specific etiological factors are not well elucidated.^5^, ^6^ Unlike cutaneous and uveal melanomas, molecular studies of mucosal melanomas show higher rates of KIT mutations, followed by NRAS and BRAF mutations. Our patient is a non‐smoker and also does not have any exposure to formaldehyde.

Most of the mucosal melanomas often occur in the occult site and present late in the course of the disease.^7^ However, they are of aggressive behavior in comparison with their cutaneous and uveal counterparts resulting in rapid progression of symptoms and early metastasis. Our patient presented with features of nasal obstruction and blood‐stained nasal discharge for one and half months. The mass is infiltrating the surrounding bones and adjacent structures in the CT scan.

Macroscopically mucosal melanomas show polypoidal growth of black‐colored and friable to grayish and firm mass. Amelanotic presentation is more common in sinonasal type (20%–25%) compared to cutaneous type (1.8%–8.1%).^8^ Our patient had grayish discoloration of the mass originating from the right maxillary antrum and protruding into the right nasal cavity.

Imaging including CT of local bones and MRI can be helpful to evaluate tumor extension. CT with 3D facial reconstruction is especially helpful in patients planned for facial reconstruction. A spontaneous high‐intensity signal on T1 with a low‐intensity signal on T2 is a typical brain and facial MRI finding. MRI also helps to differentiate between sinonasal mucosal melanoma and paranasal sinus fluid retention, delineate the borders of invasion, and detect any brain metastases. Furthermore, chest, abdomen, and pelvis CT and PET scan help stage the tumor and thereby helps management.^9^ Our patient did not have evidence of metastasis in the head and neck region. Abdominal and chest CT scans were not done.

Histologically, it is a challenging task to diagnose mucosal melanomas in absence of melanin pigmentation, and thus, immunohistochemical stain helps to reach the diagnosis. There are different morphological types of tumor cells ranging from spindle to epithelioid to plasmacytoid. Immunohistochemically, S100 and HMB‐45 are positive even in tumor cells lacking melanin pigment.^10^ However, cytokeratin is absent. Our patient had epithelioid‐type cells on microscopy with tumor cells showing positivity with HMB‐45 stain.

Sometimes, the sinonasal mucosal melanoma could be a presentation of the metastatic spectrum of the melanoma elsewhere, which changes the staging of the disease. American Joint Committee on Cancer staging manual eight editions has mentioned that all head and neck melanoma should be kept in the T3–T4 category irrespective of the size of the mass.^3^ The overall prognosis is decreased if there is any distant metastasis and advanced age. Our patient is an adult female and did not have any metastasis.

Wide surgical resection is widely accepted as the first‐line treatment of head and neck mucosal melanomas until we can resect the tumor with a negative margin. The role of adjuvant radiation therapy can be considered if the tumor can be resected completely. As mucosal melanomas are poorly radiosensitive, radiotherapy is reserved for tumors with positive surgical margins, local recurrence, locally advanced tumors, and for patients who refuse surgery or sometimes as palliative therapy. In patients with surgical treatment failure, metastatic disease, or palliation, chemotherapy can be used. The use of immunotherapy as a monotherapy or adjunct therapy in combination with other treatments is still under evaluation. Considering the poor outcome and financial status, our patient refused to undergo any sort of surgery and is under radiotherapy. She is planned for evaluation after completion of radiotherapy.

Despite aggressive therapy, the prognosis of mucosal melanomas is poor with 5‐year survival rates of 12% to 30%.^11^ A ten‐year study on sinonasal mucosal melanoma by Wang et al. including 36 cases of sinonasal mucosal melanoma has shown that the 1‐year OS was 80.6%, the 3‐year OS was 36.1%, and the 5‐year OS was 13.9%.^12^ The risk of local recurrence ranges from 37% to 54%, and metastases are found in around 50% of cases.^13^ The head and neck melanomas recur within a median time of 6 to 12 months.^14^ Negative resection margin is the most important prognostic factor. The prognosis of the disease depends on obstructive symptoms, advanced age, tumor size, vascular invasion, location, high mitotic count, marked cellular pleomorphism, and regional and distant metastasis. Other poor prognostic factors include advanced stage, old age, and tumor size greater than 3–4 cm.^15^

## 4 CONCLUSION

Primary sinonasal mucosal melanoma of the nose is an extremely rare entity, and it must be differentiated from the other tumors of the nose and paranasal sinuses. Due to its highly aggressive nature and its tendency to metastasize early, it should be diagnosed at the earliest and surgically resected promptly to increase the chances of survival. Hence, patients with minor nasal symptoms should also be assessed thoroughly to rule out any neoplastic etiologies.

## CONFLICT OF INTEREST

None.

## AUTHOR CONTRIBUTION

Kamal Gautam, Sansar Babu Tiwari, and Suraj Shrestha involved in concept and case selection. Prabin Gaire, Malati Dulal, and Bibek Man Shrestha involved in writing and collecting patient details. Roman Dhital and Arun Kumar Baral involved in editing and final draft preparation. Usha Manandhar served as a senior reviewer and involved in editing.

## ETHICAL APPROVAL

Not applicable.

## CONSENT

Written informed consent was obtained from the patient before the submission of the report. The signed Institutional Consent Form is on file.

## Data Availability

The data that support the findings of this study are available from the corresponding author upon reasonable request.
